# Spatial and Temporal Variability and Driving Factors of Carbon Dioxide and Nitrous Oxide Fluxes in Alpine Wetland Ecosystems

**DOI:** 10.3390/plants11212823

**Published:** 2022-10-24

**Authors:** Bing Yu, Wenjing Xu, Linlu Yan, Heng Bao, Hongxian Yu

**Affiliations:** College of Wildlife and Protected Area, Northeast Forestry University, Harbin 150040, China

**Keywords:** greenhouse gas fluxes, island forest, physico-chemical variables, vegetation type, wetland ecosystem

## Abstract

Plants regulate greenhouse gas (GHG) fluxes in wetland ecosystems, but the mechanisms of plant removal and plant species that contribute to GHG emissions remain unclear. In this study, the fluxes of carbon dioxide (CO_2_) and nitrous oxide (N_2_O) were measured using the static chamber method from an island forest dominated by two different species, namely *Betula platyphylla* (BP) and *Larix gmelinii* (LG), in a marsh wetland in the Great Xing’an Mountains. Four sub-plots were established in this study: (1) bare soil after removing vegetation under BP (SBP); (2) bare soil after removing vegetation under LG (SLG); (3) soil with vegetation under BP (VSBP); and (4) soil with vegetation under LG (VSLG). Additionally, the contributions of the dark respiration from plant aerial parts under BP (VBP) and LG (VLG) to GHG fluxes were calculated. We found that the substantial spatial variability of CO_2_ fluxes ranged from −25.32 ± 15.45 to 187.20 ± 74.76 mg m^−2^ h^−1^ during the study period. The CO_2_ fluxes decreased in the order of SBP > VSLG > VSBP > SLG > VLG > VBP, indicating that vegetation species had a great impact on CO_2_ emissions. Particularly, the absence of vegetation promoted CO_2_ emission in both BP and LG. Additionally, CO_2_ fluxes showed dramatically seasonal variations, with high CO_2_ fluxes in late spring (May) and summer (June, July, and August), but low fluxes in late summer (August) and early autumn (September). Soil temperatures at 0–20 cm depth were better predictors of CO_2_ fluxes than deeper soil temperatures. N_2_O fluxes were varied in different treatments with the highest N_2_O fluxes in SLG and the lowest N_2_O fluxes in VBP. Meanwhile, no significant correlation was found between N_2_O fluxes and air or soil temperatures. Temporally, negative N_2_O fluxes were observed from June to October, indicating that soil N_2_O fluxes were reduced and emitted as N_2_, which was the terminal step of the microbial denitrification process. Most of the study sites were CO_2_ sources during the warm season and CO_2_ sinks in the cold season. Thus, soil temperature plays an important role in CO_2_ fluxes. We also found that the CO_2_ flux was positively related to pH in a 10 cm soil layer and positively related to moisture content (MC) in a 50 cm soil layer in VSBP and VSLG. However, the CO_2_ flux was negatively related to pH in a 30 cm soil layer in SBP and SLG. Our findings highlight the effects of vegetation removal on GHG fluxes, and aid in the scientific management of wetland plants.

## 1. Introduction

The gas of CO_2_, an important component of greenhouse gases in the atmosphere, contributes to approximately 63% of global warming [1,2]. Along with CO_2_, N_2_O has a disproportional effect on global warming, which is potentially 298 times greater than that of CO_2_ in a 100-year time frame [2]. As the vital parts of greenhouse gases, mean CO_2_ and N_2_O has increased by 40% and 20%, respectively, since pre-industrial times on a global scale [2,3]. These rapid increases in the main greenhouse gas (CO_2_ and N_2_O) have been mainly attributed to land use changes, fossil fuel uses, and agricultural activities [4]. Although wetlands cover a small percentage of the land surface, they have a great influence on the dynamics and cycles of CO_2_ and N_2_O in nature [5,6]. Therefore, strengthening research on CO_2_ and N_2_O emissions in wetland ecosystems is of great significance for global climate change.

Studies that have been conducted on CO_2_ and N_2_O fluxes from natural wetlands worldwide [5,7,8], including estuarine tidal marshes with varying salinity [6], temperate and tropical wetlands [9], and boreal and subarctic wetlands [10], indicate that CO_2_ flux was higher during the warm growing season because of high temperatures and high aboveground biomass. The spatio-temporal CO_2_ and N_2_O fluxes varied obviously within one wetland and among different wetlands [11,12]. The temporal variations of CO_2_ and N_2_O fluxes were primarily driven by soil temperature, moisture, and water level. By contrast, the spatial variations of CO_2_ and N_2_O were mainly influenced by vegetation composition [13,14,15]. Liu et al. (2017) [14] reported a remarkably higher N_2_O production in palustrine wetlands compared with the riverine and lacustrine wetlands because of high denitrification rates. Xu et al. (2014) [6] found that the spatial variations of CO_2_ and N_2_O fluxes were primarily influenced by vegetation types. However, few studies have been conducted to investigate the spatio-temporal CO_2_ and N_2_O gas emissions in alpine wetland ecosystems. 

Given the highly heterogeneous nature due to vegetation types and climate change, the spatio-temporal changes of CO_2_ and N_2_O fluxes are uncertain in the Nanweng River Wetland National Nature Reserve (NRWNNR). The objectives of this study were to: (1) investigate the spatial and temporal variation of CO_2_ and N_2_O fluxes in the NRWNNR; and (2) determine the main influences of soil physico-chemical variables on the fluxes of CO_2_ and N_2_O. We hypothesized that CO_2_ and N_2_O fluxes from the wetlands would vary spatially because of the high environmental heterogeneity creating different micro-environments within the vegetation types. A clear understanding of the spatial variability and factors influencing CO_2_ and N_2_O fluxes in this important and critical environmental system is very crucial for management, and even for re-establishing, wetlands within the Great Xing’an Mountain areas.

## 2. Results

### 2.1. Seasonal Variation of CO_2_ and N_2_O Fluxes

**Figure 1:**
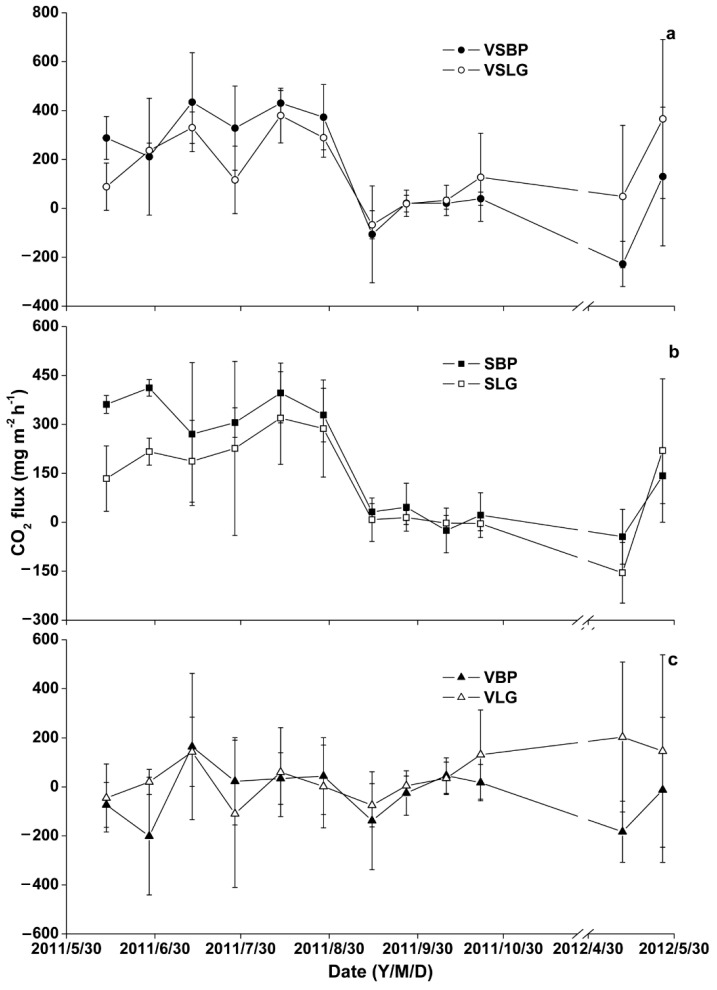
Temporal variation of CO_2_ fluxes from bare soil after removing vegetation under BP, abbreviated as SBP, bare soil after removing vegetation under LG, abbreviated as SLG, soil with vegetation under BP, abbreviated as VSBP, soil with vegetation under LG, abbreviated as VSLG, fluxes from the vegetation under BP, abbreviated as VBP, and under LG, abbreviated as VLG. (**a**) Temporal variation of CO_2_ fluxes from VSBP and VSLG; (**b**) temporal variation of CO_2_ fluxes from SBP and SLG; (**c**) temporal variation of CO_2_ fluxes from VBP and VLG.

During the study period, the mean CO_2_ fluxes ranged from −25.32 ± 15.45 to 187.20 ± 74.76 mg m^−2^ h^−1^. Higher mean CO_2_ fluxes of 187.20 ± 74.76 mg m^−2^ h^−1^ were observed in SBP, followed by VSLG (163.86 ± 30.12 mg m^−2^ h^−1^), VSBP (161.87 ± 16.68 mg m^−2^ h^−1^), SLG (120.83 ± 48.97 mg m^−2^ h^−1^), VLG (43.03 ± 25.35 mg m^−2^ h^−1^), and then VBP (−25.32 ± 15.45 mg m^−2^ h^−1^). Figure 1 shows the temporal variation of the CO_2_ fluxes measured during the study period. One-way ANOVA revealed a significant temporal variability of CO_2_ fluxes at all sites during the study period. The CO_2_ fluxes in VSLG and VSBP depicted an almost similar pattern with the higher fluxes observed in late spring (May) and summer (June, July, and August), while lower fluxes were measured in late summer (August) and early autumn (September). Interestingly, CO_2_ fluxes in VSBP were relatively higher in late spring (May) and summer (June, July, and August) than those of VSLG (Figure 1a). However, in late summer and early autumn, VSLG had relatively higher CO_2_ fluxes than VSBP. An almost similar temporal CO_2_ flux pattern to that of VSBP and VSLG was observed in SBP and SLG. In SBP and SLG, high CO_2_ fluxes were measured in late spring (May) and summer (June, July, and August) and almost low fluxes (negative fluxes) in late summer and early autumn. Comparing the temporal CO_2_ fluxes between the two sites (SBP and SLG), SBP had higher fluxes than SLG. It is quite clear that the CO_2_ fluxes measured in the sites of SBP and VSBP were higher than those measured in the sites of SLG and VSLG. Contributions to fluxes from VBP and VLG showed that VBP and VLG had negative and positive mean CO_2_ fluxes, respectively. The temporal variability of CO_2_ fluxes in VBP showed that the gas fluxes were positive in the months of July and August (Figure 1c). Conversely, negative CO_2_ fluxes in VBP were measured in the months of September, October, April, and May. Unlike the VBP, in the VLG, negative CO_2_ fluxes were measured in July, while positive fluxes were observed in October, April, and May. The mean seasonal CO_2_ emissions of VSBP and VSLG were 161.87 ± 216.64 and 163.86 ± 150.79 mg m^−2^ h^−1^, respectively, and those of VBP and VLG were −25.33 ± 106.35 and 43.03 ± 96.91 mg m^−2^ h^−1^, respectively. This showed that vegetation played a minor role in the CO_2_ fluxes of the whole island forest wetland ecological system, and the CO_2_ fluxes of VBP were C sinks; however, the CO_2_ fluxes of VLG were C resources.

**Figure 2:**
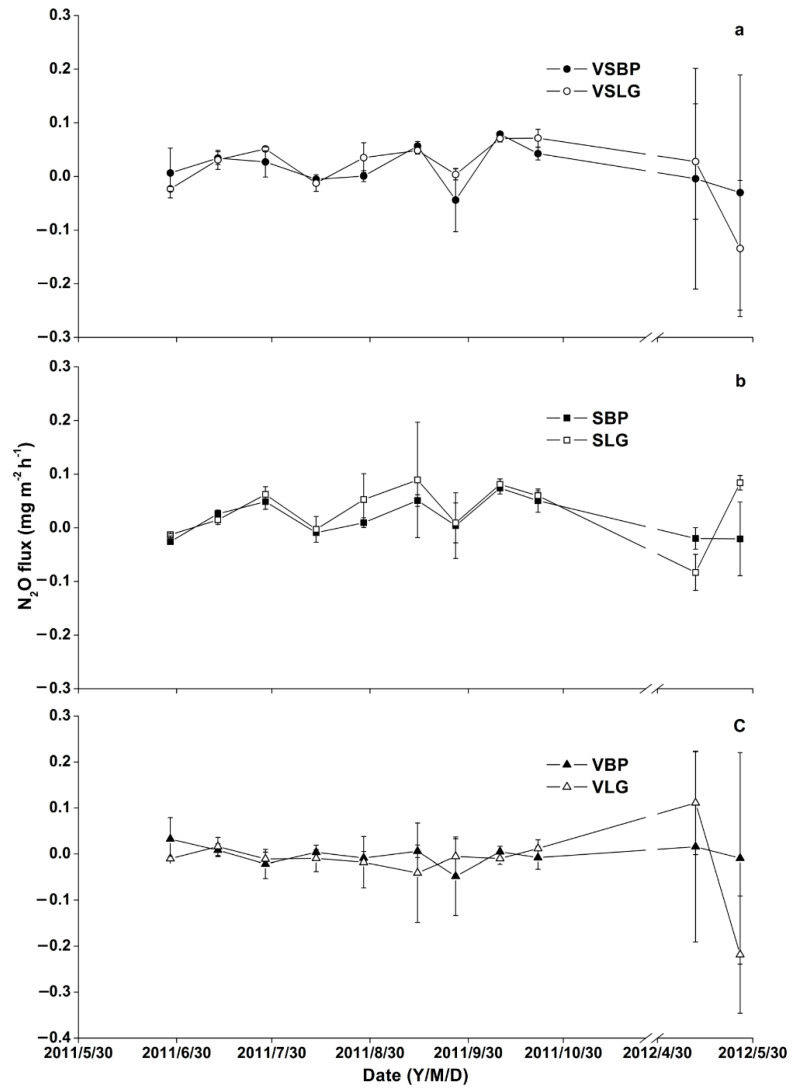
Seasonal variation of N_2_O fluxes from bare soil after removing vegetation under BP, abbreviated as SBP, bare soil after removing vegetation under LG, abbreviated as SLG, soil with vegetation under BP, abbreviated as VSBP, soil with vegetation under LG, abbreviated as VSLG, fluxes from the vegetation under BP, abbreviated as VBP, and under LG, abbreviated as VLG. (**a**) Temporal variation of N_2_O fluxes from VSBP and VSLG; (**b**) temporal variation of N_2_O fluxes from SBP and SLG; (**c**) temporal variation of N_2_O fluxes from VBP and VLG.

During the study period, the mean N_2_O fluxes ranged from −0.001 ± 0.060 to 0.032 ± 0.020 mg m^−2^ h^−1^. For the N_2_O fluxes, positive fluxes were measured in June, July, and August in VSBP and VSLG, while negative fluxes were observed in September, April, and May (Figure 2a). VSLG had relatively higher N_2_O fluxes values compared to VSBP. In SBP and SLG, the temporal pattern of N_2_O fluxes was almost similar. The fluxes were negative in the period of early June (summer), and they gradually increased positively until the end of July (summer). This was followed by slight decrease in early August, and then a gradual increase in mid-August and September. In April and May, the N_2_O fluxes were negative in both SBP and SLG (Figure 2b). As shown in Figure 2b, the temporal N_2_O fluxes in SLG were higher than those in SBP. The N_2_O fluxes from VBP and VLG were negative from June to October. However, in April, positive fluxes were observed in VLG (Figure 2c). Our results showed that the average N_2_O emissions from VSBP and SBP were 0.015 ± 0.037 and 0.017 ± 0.035 g m^−2^ d^−1^, respectively, and those from VSLG and SLG were 0.015 ± 0.059 and 0.032 ± 0.053 mg m^−2^ d^−1^, respectively. Therefore, island forest wetlands with vegetation had a lower emission rate of N_2_O than those with no vegetation.

### 2.2. Relationships between Gas Fluxes and Temperatures

Regression analysis revealed significant correlations between CO_2_ fluxes and soil temperatures at 5, 10, and 15 cm depths in VSBP and VSLG (R^2^ = 0.264–0.292; *p* < 0.01). In SBP and SLG, the relationships between CO_2_ fluxes, air temperatures, and soil temperatures at all depths were significantly correlative (R^2^ = 0.281–0.524; *p* < 0.01) (Table 1). When the level of confidence was set at 0.05, the relationships between CO_2_ fluxes, air temperatures, and soil temperatures at 0, 20, 30, and 40 cm depths in VSBP and VSLG were significantly correlative (R^2^ = 0.184–0.688; *p* < 0.05). However, there were no indications of any associations between CO_2_ fluxes and air or soil temperatures at all depths in VBP and VLG (Table 1). 

The results showed there were no significant correlations between N_2_O fluxes and air, soil temperatures in VSBP and VSLG, SBP and SLG, VBP and VLG (Table 2). These showed that temperature had little influence on N_2_O fluxes of forest in swamp wetlands in eastern Great Xing’an Mountain. 

### 2.3. Vertical Distributions and Relationships of Soil Properties

**Figure 3:**
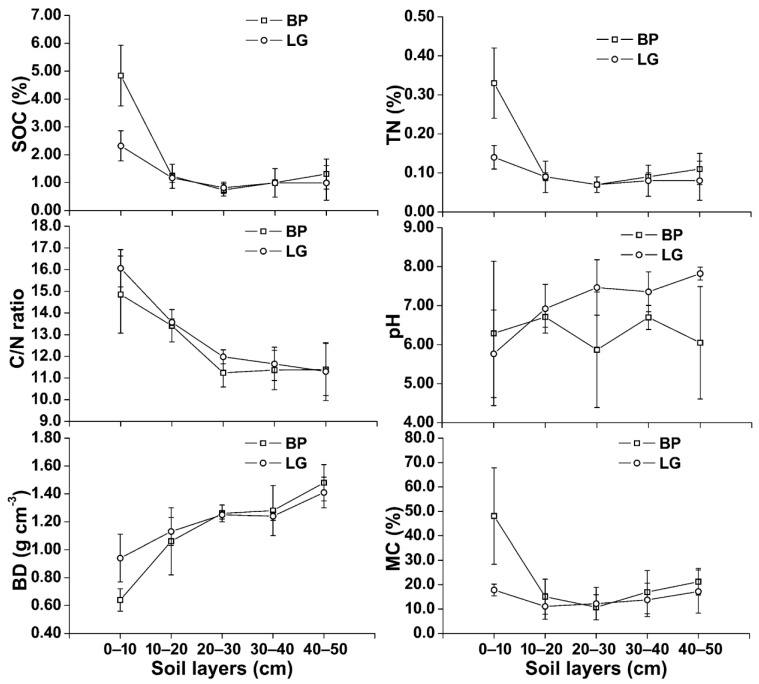
Vertical distributions of soil properties of BP and LG.

The physico-chemical properties of the soils from the study sites are shown in Figure 3 [16]. The pH of soils in BP was higher than that in LG in 0–10 cm soil depths, but this was contrary in other soil layers. The biggest differences in the SOC, TN, BD, MC, and C/N ratio of soils between BP and LG mainly existed in 0–10 cm soil layers. From 0–10 cm to 40–50 cm soil layers in the study region, SOC, TN, and MC mainly experienced drop and increase trends, and BD mainly experienced an increasing trend, but the C/N ratio mainly experienced a drop trend.

**Figure 4:**
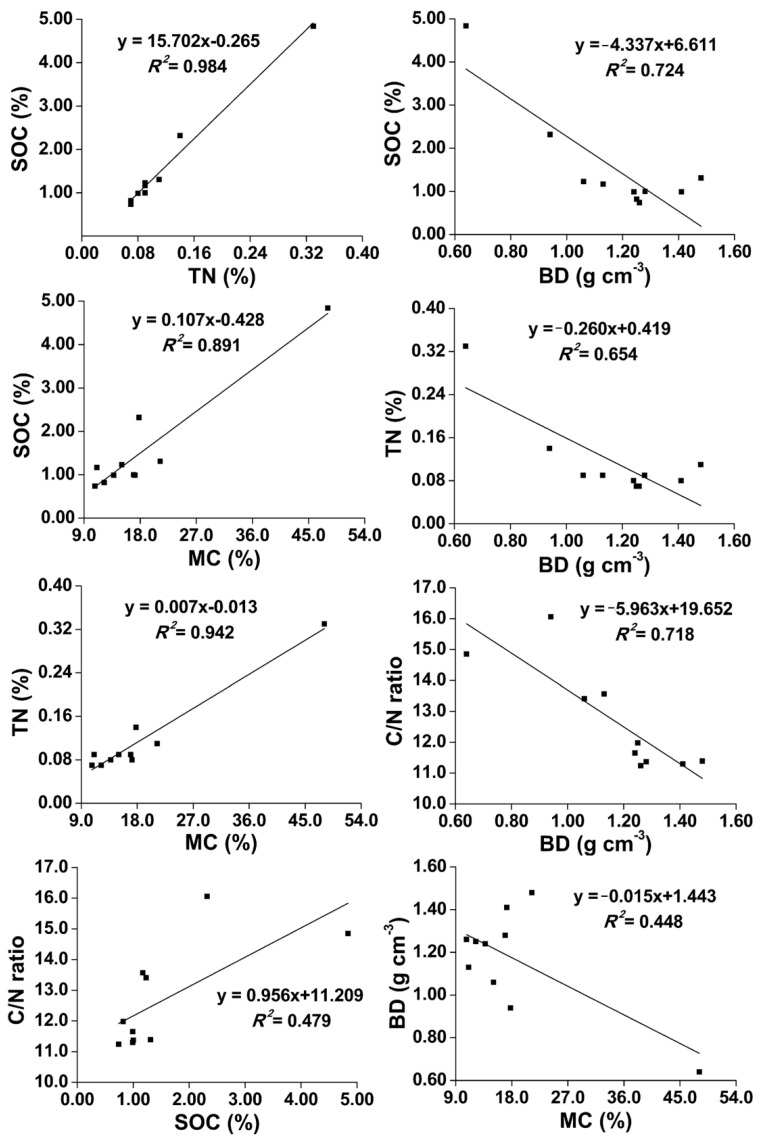
Relationships between soil properties of two island forest wetlands.

The study results indicate there were significantly positive correlations between SOC and TN, MC (*p* < 0.01), and C/N ratio (*p* < 0.05), and that TN was positively correlated with MC (*p* < 0.01) in soils of two types of forest in swamp wetlands (Figure 4). In addition, there were significantly negative correlations between BD and SOC, TN, C/N ratio (*p* < 0.01), and MC (*p* < 0.05). These results fully prove there were good correlations between the physico-chemical properties of island forest wetland soils.

### 2.4. Relationships between Gas Fluxes and Soil Properties

**Figure 5:**
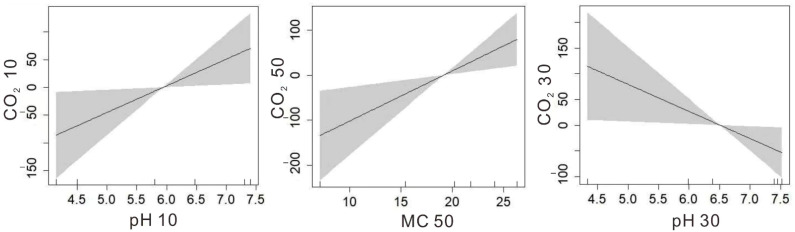
Relationships between gas fluxes and soil properties in NRWNNR. The (**left**) picture shows the relationship between CO_2_ fluxes and pH in 10 cm soil layer in VSBP and VSLG, the (**middle**) picture shows the relationship between CO_2_ fluxes and MC in 50 cm soil layer in VSBP and VSLG, and the (**right**) picture shows the relationship between CO_2_ fluxes and pH in 30 cm soil layer in SBP and SLG.

We found there were some relationships between CO_2_ fluxes and soil properties, and influence factors were different in different soil layers (Figure 5). In VSBP and VSLG, CO_2_ fluxes were positively related to pH in a 10 cm soil layer (R^2^ = 0.146; *p* = 0.037), and there was a significantly positive relationship between CO_2_ fluxes and MC in a 50 cm soil layer (R^2^ = 0.215; *p* = 0.013). However, in SBP and SLG, CO_2_ fluxes were negatively related to pH in a 30 cm soil layer (R^2^ = 0.142; *p* = 0.039). However, there were no significant relationships between N_2_O fluxes and soil properties. The results show that vegetation removal had a great influence on the relationships between CO_2_ fluxes and soil properties. 

## 3. Discussion

Compared with previous studies, the CO_2_ fluxes measured in the NRWNNR, which ranged from −25.32 to 187.20 mg m^−2^ h^−1^, were lower than those measured in the wetlands of a montane permafrost region, northeast China (403.47 mg m^−2^ h^−1^) [17]. However, note that Liu et al. [17] conducted their study in a montane permafrost region in the Great Xing’an Mountains during the thawing seasons. The rates of N_2_O fluxes in our study were in the same range to those measured in the Daxing’an Mountains (−0.0 14 to 0.013 mg m^−2^ h^−1^) [18] and the Qinghai–Tibetan Plateau (−0.022 to 0.014 mg m^−2^ h^−1^) [19], but lower than those from the wetlands in permafrost in northeast European Russia (0.0021–0.092 mg m^−2^ h^−1^) [20], subarctic East European tundra (0.079–0.129 mg m^−2^ h^−1^) [21], and the Eboling Mountain (1.286–2.662 mg m^−2^ h^−1^) [22]. 

Consistent with previous studies [11,23,24], we found high spatio-temporal variations in CO_2_ and N_2_O fluxes. The forest vegetation species of BP had relatively higher CO_2_ fluxes than that of LG. The differences of CO_2_ fluxes among the sites could be likely explained by the differences in SOC and biomass. The forest dominated by BP had a relatively higher SOC and biomass compared to the forest dominated by LG. While assessing the factors influencing CO_2_ emissions from wetlands in the Liaohe Delta, northeast China, Olsson et al. (2015) [25] observed that SOC and biomass strongly impacted gas emissions. Although no significant influence was observed of SOC on CO_2_ fluxes, about 51.2% of CO_2_ fluxes were explained by SOC in the top 20–30 cm soil depth. It is quite possible that the higher decomposition of SOC and litter that happened in the sites dominated by BP lead to relatively higher CO_2_ fluxes. It is documented that the quality and quantity of SOC is mainly determined by the vegetation types and species, which influences soil respiration and decomposition [26]. 

The temporal pattern of CO_2_ fluxes was closely related to the soil temperatures at all sites, which suggested that temperature was one of the main controlling factors for ecosystem respiration [27,28,29,30,31]. This concurs with the findings in other studies [25,32]. In this study, the CO_2_ fluxes appeared to reach their peak in summer (June, July, and August) and then decreased in October, April, and May during the growth cessation period, which might attribute their variations over time to soil temperatures and, to some extent, to differences in biomass [25]. Soil temperature was the dominant environmental factor in controlling CO_2_ fluxes in cold-temperate wetland ecosystems [17,33]. Additionally, the activities of microorganisms are generally temperature-dependent in cold-temperate wetland ecosystems. We also found there were strong positive relationships between CO_2_ fluxes and soil temperatures at each depth in VSBP and VSLG, and SBP and SLG (Table 1). 

Our study also revealed that soil temperatures at a 0–20 cm depth were better predictors of CO_2_ fluxes than deeper soil temperatures (Table 1). The SOC and TN usually decreased with depth in this study area, which indicated more active decompositions and exchanges of matter and energy in the topsoil layer [16]. Likely, the active growing roots of the vegetation species in this study area were abundant on the topsoil layer, which also meant more respiration. It is tempting to note the CO_2_ production from the topsoil layer (0–20 cm depth) was the main contributor to CO_2_ fluxes and were not constrained by the carbon source quantity, which led to soil temperatures being the main constraint in the study area due to the thermal energy required for CO_2_ productions. 

**Figure 6:**
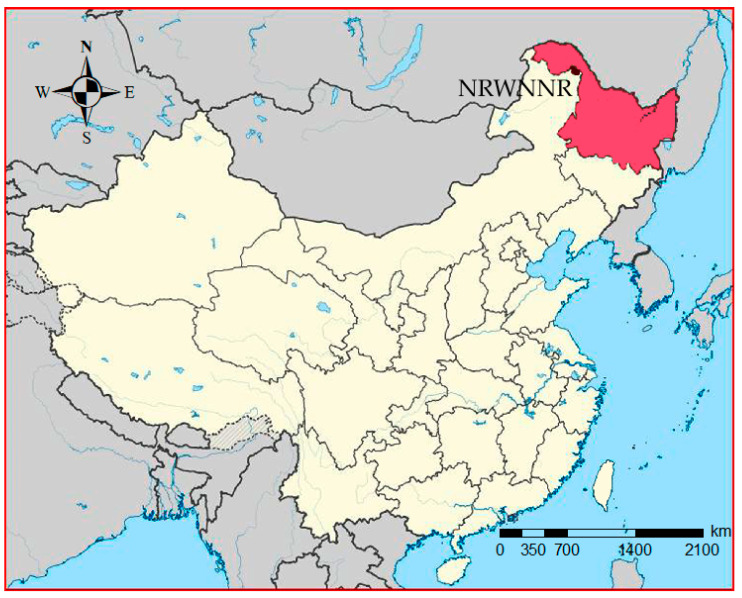
Map of the study area of NRWNNR in northeast China. Heilongjiang province in deep color, NRWNNR in the deepest color.

SOC was significantly correlated with the TN and C/N ratio for all the soils (*p* < 0.001) of two natural inland saline–alkaline wetlands in northeastern China [34]. Permanently flooded soils provide more suitable conditions to accumulate carbon, whereas intermittently flooded sites usually provide conditions for greater carbon inputs [35]. Our current study results were consistent with these conclusions (Figure 6). Wang et al. (2013) [36] reported a significantly positive linear correlation between SOC and TN (R^2^ = 0.58), and a logarithmic correlation between SOC and BD (R^2^ = 0.84). However, our study results were bigger than the former and smaller than the latter. Spearman’s rank correlation analysis showed that SOC was positively correlated with TN, and this relationship was much stronger in the freshwater-treated sites (R^2^ = 0.84, *p* < 0.05) compared to the reference sites in the Yellow River delta of China (R^2^ = 0.47, *p* < 0.05) [37]. A positive correlation was also found between SOC and MC (R^2^ = 0.38, *p* < 0.05) [37], and our study results showed that the correlation between SOC and MC was bigger than it. 

The degree of MC also regulates CO_2_ production [38]. In this study, we found there was a positive correlation between CO_2_ emissions and MC in a 50 cm soil depth. Extremely dry or wet conditions can hamper aerobic microbial activity and reduce CO_2_ emissions [39]. The water levels were all deeper than 40 cm in all sampling sites in this study. Therefore, the activities of aerobic microorganisms were hindered by extreme dry conditions, which led to a reduction in CO_2_ emissions. The spatial variation of soil CO_2_ emissions in the field significantly correlated with the soil pH, explaining up to 24% of the variability [40]. In VSBP and VSLG, CO_2_ fluxes were positively related to pH in a 10 cm soil layer, which was in agreement with the study of Sauze et al. (2017) [41]. However, in SBP and SLG, CO_2_, fluxes were negatively related to pH in a 30 cm soil layer. This could be the due to the vegetation removal, which influenced the relationship between CO_2_ fluxes and pH.

For N_2_O fluxes, sites with vegetation under BP and LG had relatively lower fluxes than the sites with bare soils, which was contrary to the findings in other studies. For example, while assessing the emissions of N_2_O from constructed wetlands in Europe, Søvik et al. (2006) [42] observed high fluxes of N_2_O in vegetated sites compared to bare soil sites. Hernandez and Mitsch (2006) [43] also observed high N_2_O fluxes from highly vegetated marsh plots when the plots were more inundated than bare soil sites in created riparian marsh wetlands. Likely, the variation of hydrological conditions in this study cases could have led to these different observations. The plant parenchymal system under flooding conditions is more active in transporting oxygen from the shoots to the roots, and probably in transporting gases from the soil to the atmosphere, than that under exposed conditions [43]. 

In addition, negative N_2_O fluxes in June to October indicated that soil N_2_O fluxes were reduced and emitted as N_2_, which was the terminal step of the microbial denitrification process [44]. Interestingly, positive N_2_O fluxes were observed during spring (April and May) (Figure 2). The positive fluxes during the spring period were in agreement with the previous study [45]. Teepe et al. (2000) [45] attributed N_2_O fluxes in spring to the physical releases of trapped N_2_O and/or, to the denitrification in the freeze–thaw period. The emission of N_2_O from the soil is controlled by the soil temperature and nitrogen availability [46]. Hernandez and Mitsch (2006) [43] also found a strong influence of temperature on N_2_O emissions, with high fluxes during summers with high soil temperatures (≥ 20 °C). The correlation coefficients of seasonal N_2_O fluxes to soil temperatures in the non-waterlogged and seasonally waterlogged freshwater marsh of *Deyeuxia angustifolia* in northeast China were 0.660 and 0.534, respectively. This highly significant correlation is likely due to the fact that an increase in soil temperature positively influences microbiological activities and gas diffusion, whereas it negatively affects the solubility of N_2_O. Additionally, N_2_O emissions increasing with temperature is not only the function of temperature-produced N_2_O by the microbial process, but also of temperature-induced N_2_O solubility [47]. However, no temperature-related seasonal trends were found in the temporal variation of the N_2_O fluxes when soil temperatures varied from 5 to 15 °C in a vegetated-riparian-buffer zone in Belgium [48]. Other research results also showed that N_2_O emission flux was not related to soil temperature [49,50]. We also found there were no significant correlations between N_2_O fluxes and soil temperatures in all sites. The reason for this could be that the soil temperatures were almost below 15 °C instead of above 20 °C in all sites, and thus the soil temperature could not positively influence microbiological activity and gas diffusion. 

## 4. Materials and Methods 

### 4.1. Study Area

The NRWNNR lies at 125°07′55″ E–125°50′05″ E, 51°05′07″ N–51°39′24″ N, covering approximately 1478 km^2^ of wetlands in the Great Xing’an Mountains Areas (Figure 6) [16]. The NRWNNR is under the influence of a cold-temperate semi-humid monsoon climate with an annual average temperature of −5 °C to −1 °C and a mean annual precipitation of 390–490 mm [17]. The NRWNNR houses the largest cold-temperate wetland ecosystem in China. The wetlands in the NRWNNR consist mostly of swamp, including moss bog, shrub swamp (swamp with at least 30% shrub cover), meadow bog, and forest bog (bog with at least 20% tree cover) [16,51,52]. The vegetation in this area belongs to the southern *Quercus mongolica* and *Larix gmeliniii* forest region in the Great Xing’an Mountains’ vegetation division [53]. The island forest wetland is a typical wetland type in this region. The vegetation of island forest in this area is classified into two types based on the dominant vegetation species [54]. The forest of BP is dominated by *B. platyphylla* and interspersed with *Rosa acicularis*, *Rhododendron basilicum*, *Alnus glutinosa*, and *Cyperus rotundus*, while the forest of LG is dominated by *Larix gmeliniii* and interspersed with *Ribes sativum* and *Cyperus rotundus* [54]. The main soil types are peat bog soil, peat soil, and meadow swamp soil. This wetland is an important water source for a population of over 10 million in the Nenjiang Basin, while also ensuring the recharge of 350 million m^3^ of water for the Zhalong Nature Reserve per year [51]. 

### 4.2. Experiment Design

**Figure 7:**
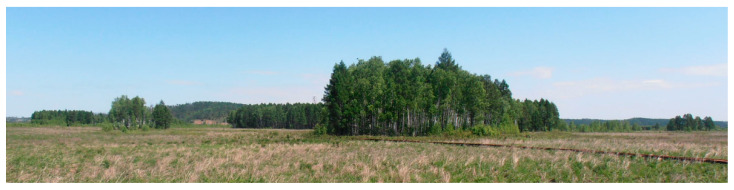
Map of the objects of study in NRWNNR.

This study employed the use of a representative sample plot which was selected in each wetland with different vegetation types (Figure 7) [16]. In each site, sub-plot sampling was randomly established to examine whether the existence of vegetation affected the CO_2_ and N_2_O fluxes. Three replicated sub-plots were measured at each sampling site and an opaque chamber (0.5 m × 0.5 m × 0.5 m) made of stainless steel, as well as two 0.5 m × 0.5 m × 0.5 m steel bases, were used for head-space sampling at each plot; in one base, the vegetation was left, but in the other base, the vegetation was removed to ground level. In order to reduce temperature fluctuation within the gas sampling systems, the chambers were shaded with Styrofoam [17]. Moreover, fans were installed inside the chambers to keep the air mixed. Only during the sampling periods were the chambers placed into square bases and the joints made airtight with water to prevent gas leakage.

### 4.3. Environmental Variables and Soil Sampling

At each plot in a sampling site, the air temperature and temperature in the chambers were measured by digital thermometers (JM624, China) during the CO_2_ and N_2_O flux sampling period. Additionally, soil temperature at depths of 0, 5, 10, 15, 20, 30, and 40 cm was measured using precise geothermometers. A well was dug to determine the water level in each plot, but the water levels were all deeper than 40 cm, so the water level was not considered as a variable.

In August, from each sampling site, three intact soil cores were collected to a depth of 50 cm, and then profiled at 10 cm intervals (0–10 cm, 10–20 cm, 20–30 cm, 30–40 cm, and 40–50 cm). The collected soil samples were taken to the laboratory and air-dried for three weeks. Visible plant roots/litter and stones were removed from the dried soils. Each soil sample was mixed thoroughly, grounded, and sieved through a 100-mesh sieve. Around 0.05 g of grounded and sieved soil was placed in a desiccator with a beaker of concentrated hydrochloric acid for 24 h in order to remove carbonates [55]. Soil organic carbon (SOC) and total nitrogen (TN) were determined using an automatic elemental analyzer (Flash EATM 1112, Italy), using 130 and 100 mL min^−1^ of He and O_2_ and an oven temperature of 50 °C [56]. All determinations were made in triplicate. Soil pH was measured with a pH analyzer (IQ35, America). Soil bulk density (BD) and MC were calculated on a dry-weight basis.

### 4.4. CO_2_ and N_2_O Flux Measurements

The CO_2_ and N_2_O fluxes were measured bimonthly from 10:00 am to 12:00 am during the daytime at each site between June and October in 2011, and in May 2012. A sample was taken every 10 min using a 60 mL syringe within 30 min. The CO_2_ and N_2_O concentrations were examined by a gas chromatography unit (7820A GC system, Agilent Technologies Inc., Santa Clara CA, USA), equipped with both flame ionization and electron capture detectors in the Laboratory of Northeast Institute of Geography and Agroecology, Chinese Academy of Sciences. Then, the gas fluxes (J) were calculated using the gradient of the time-series of the sampled gas concentrations and the calculation equation as follow [57]:

$$J=\frac{dc}{dt}\frac{M}{V_{0}}\frac{P}{P_{0}}\frac{T_{0}}{T}H$$

where d*c*/d*t* is the curve slope of the temporal variation in the gas concentration, *M* (g mol^−1^) is the gas molar mass, *P* (Pa) is the atmospheric pressure at the sampling site, *T* (K) is the absolute temperature during sampling period, and H (m) is the chamber’s height. *V*_0_ (L mol^−1^), *P*_0_ (Pa), and *T*_0_ (K) are the gas molar volume, standard pressure, and standard temperature (International Union of Pure and Applied Chemistry), respectively. Flux attributable to a conduit provided by the vegetation, or to the aboveground vegetation itself, was calculated via subtraction for the VBP and VLG sites.

### 4.5. Statistical Analysis

One-way ANOVA was used to see whether the existence of vegetation types had significant effects on CO_2_ and N_2_O fluxes. Linear regression was conducted to assess the relationships between gas (CO_2_ and N_2_O) fluxes, air temperature, and soil temperatures. A GAM model was used to test the relationships between CO_2_ fluxes, N_2_O fluxes, and soil physico-chemical variables. All of the statistical analyses were performed by SPSS STATISTICS 19.0 and R 4.1.3 software, and figures were drawn by OriginPro 8.0 software. 

## 5. Conclusions

The results showed that there were differences in CO_2_ and N_2_O fluxes in different island forest ecosystems. Fluxes varied between the different vegetation covers, and plant presence or absence had an important role in GHG emissions. The NRWNNR acted as a CO_2_ source in May to August and as a CO_2_ sink in April. Moreover, the NRWNNR acted as an N_2_O sink in June to October and as an N_2_O source in April. We were able to identify several environmental parameters that influence CO_2_ and N_2_O fluxes. CO_2_ fluxes were closely related to soil temperature at all sites, which suggested that temperature was one of the main controlling factors for ecosystem respiration. Even under the same vegetation cover, CO_2_ and N_2_O fluxes varied with air and soil temperatures, MC, and pH. Our findings highlight the effect of vegetation removal to GHG fluxes, and aid in the scientific management of wetland plants.

## Figures and Tables

**Table 1:** Relationships between CO_2_ fluxes and air and soil temperatures within the different vegetation types within NRWNNR.

Sites	Equations	Variables	Ranges for the Variables	R^2^	*p*
VSBP and VSLG	F = 19.183T − 184.912	T at air	9.6–23.4 °C	0.688	0.041 *
	F = 16.546T − 147.717	T at 0 cm depth	8.6–25.7 °C	0.685	0.042 *
	F = 21.605T − 35.369	T at 5 cm depth	2.0–17.3 °C	0.292	0.006 **
	F = 21.571T − 10.967	T at 10 cm depth	2.1–15.4 °C	0.280	0.008 **
	F = 21.337T + 3.460	T at 15 cm depth	0.8–14.1 °C	0.264	0.010 **
	F = 21.182T + 9.998	T at 20 cm depth	0.0–13.5 °C	0.251	0.013 *
	F = 20.323T + 23.991	T at 30 cm depth	−0.2–12.5 °C	0.217	0.022 *
	F = 19.623T + 37.116	T at 40 cm depth	−0.4–11.8 °C	0.184	0.037 *
SBP and SLG	F = 10.986T − 45.153	T at air	3.6–28.0 °C	0.281	0.008 **
	F = 12.525T − 63.982	T at 0 cm depth	1.6–31.9 °C	0.422	0.001 **
	F = 22.373T − 73.816	T at 5 cm depth	1.0–18.7 °C	0.507	0.000 **
	F = 25.908T − 67.062	T at 10 cm depth	1.8–16.3 °C	0.524	0.000 **
	F = 25.993T − 48.193	T at 15 cm depth	0.7–14.7 °C	0.496	0.000 **
	F = 24.910T − 31.465	T at 20 cm depth	0.0–14.0 °C	0.452	0.000 **
	F = 23.920T − 12.433	T at 30 cm depth	−0.2–12.9 °C	0.385	0.001 **
	F = 22.760T + 6.263	T at 40 cm depth	−0.4–12.1 °C	0.318	0.004 **
VBP and VLG	F = −1.676T + 39.228	T at air	3.6–28.0 °C	0.015	0.565
	F = −1.363T + 34.439	T at 0 cm depth	2.4–33.2 °C	0.013	0.602
	F = −2.445T + 31.283	T at 5 cm depth	2.0–17.3 °C	0.011	0.623
	F = −2.486T + 28.883	T at 10 cm depth	2.1–15.4 °C	0.011	0.623
	F = −2.361T + 26.490	T at 15 cm depth	0.8–14.1 °C	0.010	0.647
	F = −2.603T + 27.633	T at 20 cm depth	0.0–13.5 °C	0.011	0.620
	F = −2.331T + 24.781	T at 30 cm depth	−0.2–12.5 °C	0.009	0.667
	F = −2.014T + 21.757	T at 40 cm depth	−0.4–11.8 °C	0.006	0.723

F indicates CO_2_ fluxes, T indicates temperatures. * and ** indicate significance at the 0.05 and 0.01 levels, respectively.

**Table 2:** Relationships between N_2_O fluxes, air and soil temperatures within the different vegetation types within NRWNNR.

Sites	Equations	Variables	Ranges for the Variables	R^2^	*p*
VSBP and VSLG	F = −0.0025T + 0.0583	T at air	9.6–23.4 °C	0.258	0.303
	F = −0.0025T + 0.0567	T at 0 cm depth	8.6–25.7 °C	0.301	0.259
	F = 0.0004T + 0.0108	T at 5 cm depth	2.0–17.3 °C	0.002	0.844
	F = 0.0013T + 0.0042	T at 10 cm depth	2.1–15.4 °C	0.016	0.577
	F = 0.0033T − 0.0129	T at 15 cm depth	3.8–12.5 °C	0.159	0.433
	F = 0.0038T − 0.0154	T at 20 cm depth	3.4–12.1 °C	0.205	0.367
	F = 0.0046T − 0.0196	T at 30 cm depth	2.7–11.4 °C	0.290	0.271
	F = 0.0054T − 0.0221	T at 40 cm depth	2.3–10.7 °C	0.353	0.214
SBP and SLG	F = −0.0021T + 0.0633	T at air	3.6–28.0 °C	0.148	0.077
	F = −0.0013T + 0.0458	T at 0 cm depth	1.6–31.9 °C	0.058	0.280
	F = −0.0008T + 0.0342	T at 5 cm depth	1.0–18.7 °C	0.013	0.620
	F = 0.0004T + 0.0225	T at 10 cm depth	1.8–16.3 °C	0.001	0.915
	F = 0.0008T + 0.0167	T at 15 cm depth	0.7–14.7 °C	0.011	0.646
	F = 0.0013T + 0.0154	T at 20 cm depth	0.0–14.0 °C	0.015	0.589
	F = 0.0029T + 0.0004	T at 30 cm depth	2.5–11.8 °C	0.159	0.434
	F = 0.0042T − 0.0038	T at 40 cm depth	1.9–11.0 °C	0.239	0.326
VBP and VLG	F = 0.0017T − 0.0379	T at air	3.6–28.0 °C	0.051	0.311
	F = 0.0017T − 0.0363	T at 0 cm depth	2.4–33.2 °C	0.054	0.298
	F = 0.0013T − 0.0213	T at 5 cm depth	2.0–17.3 °C	0.012	0.631
	F = 0.0008T − 0.0167	T at 10 cm depth	4.9–13.4 °C	0.043	0.693
	F = 0.0008T − 0.0167	T at 15 cm depth	3.8–12.5 °C	0.046	0.684
	F = 0.0008T − 0.0167	T at 20 cm depth	3.4–12.1 °C	0.051	0.666
	F = 0.0008T − 0.0167	T at 30 cm depth	2.7–11.4 °C	0.050	0.670
	F = 0.0008T − 0.0163	T at 40 cm depth	2.3–10.7 °C	0.047	0.680

F indicates N_2_O fluxes, T indicates soil temperatures.

## References

[1] Houghton J.T., Ding Y., Griggs J., Noguer M., Johnson C.A. (2001). Climate Change 2001: The Scientific Basis.

[2] Stocker T.F., Qin D., Plattner G.-K., Tignor M.M.B., Allen S.K., Boschung J., Nauels A., Xia Y., Bex V., Midgley P.M., IPCC (2013). Climate Change 2013: The Physical Science Basis. Contribution of Working Group I to the Fifth Assessment Report of the Intergovernmental Panel on Climate Change.

[3] Dobbie K.E., Smith K.A. (2006). The effect of water table depth on emissions of N_2_O from a grassland soil. Soil Use Manag..

[4] Mazzetto A., Barneze A., Feigl B., Van Groenigen J., Oenema O., Cerri C. (2014). Temperature and moisture affect methane and nitrous oxide emission from bovine manure patches in tropical conditions. Soil Biol. Biochem..

[5] Mwagona P.C., Yao Y.L., Shan Y.Q., Yu H.X. (2019). Greenhouse gas emissions from intact riparian wetland soil columns continuously loaded with nitrate solution: A laboratory microcosm study. Environ. Sci. Pollut. R.

[6] Xu X.W., Zou X.Q., Cao L.G., Zhamangulova N., Zhao Y.F., Tang D.H., Liu D.W. (2014). Seasonal and spatial dynamics of greenhouse gas emissions under various vegetation covers in a coastal saline wetland in southeast China. Ecol. Eng..

[7] Beringer J., Livesley S.J., Randle J., Hutley L.B. (2013). Carbon dioxide fluxes dominate the greenhouse gas exchanges of a seasonal wetland in the wet–dry tropics of northern Australia. Agric. For. Meteorol..

[8] Juszczak R., Augustin J. (2013). Exchange of the greenhouse gases methane and nitrous oxide between the atmosphere and a temperate peatland in central Europe. Wetlands.

[9] Couwenberg J., Dommain R., Joosten H. (2010). Greenhouse gas fluxes from tropical peatlands in south-east Asia. Glob. Chang. Biol..

[10] Ström L., Christensen T.R. (2007). Below ground carbon turnover and greenhouse gas exchanges in a sub-arctic wetland. Soil Biol. Biochem..

[11] Jacinthe P., Bills J., Tedesco L., Barr R. (2012). Nitrous oxide emission from riparian buffers in relation to vegetation and flood frequency. J. Environ. Qual..

[12] Jørgensen C.J., Struwe S., Elberling B. (2011). Temporal trends in N_2_O flux dynamics in a Danish wetland—Effects of plant-mediated gas transport of N2O and O2 following changes in water level and soil mineral-N availability. Glob. Chang. Biol..

[13] Dinsmore K.J., Skiba U.M., Billett M.F., Rees R.M. (2009). Effect of water table on greenhouse gas emissions from peatland mesocosms. Plant Soil.

[14] Liu Y., Liu G.H., Xiong Z.J., Liu W.Z. (2017). Response of greenhouse gas emissions from three types of wetland soils to simulated temperature change on the Qinghai-Tibetan Plateau. Atmos. Environ..

[15] Sun Z.G., Wang L.L., Tian H.Q., Jiang H.H., Mou X.J., Sun W.L. (2012). Fluxes of nitrous oxide and methane in different coastal Suaeda salsa marshes of the Yellow River estuary, China. Chemosphere.

[16] Yu B., Stott P., Yu H.X., Li X.Y. (2013). Methane emissions and production potentials of forest swamp wetlands in the eastern great Xing’an Mountains, Northeast China. Environ. Manag..

[17] Liu X., Guo Y.D., Hu H.Q., Sun C.K., Zhao X.K., Wei C.L. (2015). Dynamics and controls of CO_2_ and CH_4_ emissions in the wetland of a montane permafrost region, northeast China. Atmos. Environ..

[18] Cui Q., Song C.C., Wang X.W., Shi F.X., Yu X.Y., Tan W.W. (2018). Effects of warming on N_2_O fluxes in a boreal peatland of Permafrost region, Northeast China. Sci. Total Environ..

[19] Chen X.P., Wang G.X., Zhang T., Mao T.X., Wei D., Hu Z.Y., Song C.L. (2017). Effects of warming and nitrogen fertilization on GHG flux in the permafrost region of an alpine meadow. Atmos. Environ..

[20] Voigt C., Lamprecht R.E., Marushchak M.E., Lind S.E., Novakovskiy A., Aurela M., Martikainen P.J., Biasi C. (2017). Warming of subarctic tundra increases emissions of all three important greenhouse gases-carbon dioxide, methane, and nitrous oxide. Global Change Biol..

[21] Repo M.E., Susiluoto S., Lind S.E., Jokinen S., Elsakov V., Biasi C., Virtanen T., Martikainen P.J. (2009). Large N_2_O emissions from cryoturbated peat soil in tundra. Nat. Geosci..

[22] Mu C.C., Abbott B.W., Zhao Q., Su H., Wang S.F., Wu Q.B., Zhang T.J., Wu X.D. (2017). Permafrost collapse shifts alpine tundra to a carbon source but reduces N_2_O and CH_4_ release on the northern Qinghai-Tibetan Plateau. Geophys. Res. Lett..

[23] Hefting M.M., Bobbink R., Janssens M.P. (2006). Spatial variation in denitrification and N_2_O emission in relation to nitrate removal efficiency in a N-stressed riparian buffer zone. Ecosystems.

[24] Song C.C., Yan B.X., Wang Y.S., Wang Y.Y., Lou Y.J., Zhao Z.C. (2003). Fluxes of carbon dioxide and methane from swamp and impact factors in Sanjiang Plain, China. Chin. Sci. Bull..

[25] Olsson L., Ye S., Yu X., Wei M., Krauss K.W., Brix H. (2015). Factors influencing CO_2_ and CH_4_ emissions from coastal wetlands in the Liaohe Delta, Northeast China. Biogeosciences.

[26] Nag S.K., Liu R., Lal R. (2017). Emission of greenhouse gases and soil carbon sequestration in a riparian marsh wetland in central Ohio. Environ. Monit. Assess..

[27] Yu C.Q., Wang J.W., Shen Z.X., Fu G. (2019). Effects of experimental warming and increased precipitation on soil respiration in an alpine meadow in the Northern Tibetan Plateau. Sci. Total Environ..

[28] Zhong Z.M., Shen Z.X., Fu G. (2016). Response of soil respiration to experimental warming in a highland barley of the Tibet. SpringerPlus.

[29] Shen Z.X., Wang J.W., Sun W., Li S.W., Fu G., Zhang X.Z., Zhang Y.J., Yu C.Q., Shi P.L., He Y.T. (2016). The soil drying along the increase of warming mask the relation between temperature and soil respiration in an alpine meadow of Northern Tibet. Pol. J. Ecol..

[30] Shen Z.X., Li Y.L., Fu G. (2015). Response of soil respiration to short-term experimental warming and precipitation pulses over the growing season in an alpine meadow on the Northern Tibet. Appl. Soil Ecol..

[31] Zhang X.Z., Shen Z.X., Fu G. (2015). A meta-analysis of the effects of experimental warming on soil carbon and nitrogen dynamics on the Tibetan Plateau. Appl. Soil Ecol..

[32] Krauss K.W., Whitbeck J.L., Howard R.J. (2012). On the relative roles of hydrology, salinity, temperature, and root productivity in controlling soil respiration from coastal swamps (freshwater). Plant Soil.

[33] Miao Y.Q. (2013). Net Ecosystem Carbon Fluxes of Peatland in the Continuous Permafrost Zone, Great Hinggan Mountains. Ph.D. Thesis.

[34] Bai J., Cui B., Deng W., Yang Z., Wang Q., Ding Q. (2007). Soil organic carbon contents of two natural inland saline-alkalined wetlands in northeastern China. J. Soil Water Conserv..

[35] Bernal B., Mitsch W.J. (2008). A comparison of soil carbon pools and profiles in wetlands in Costa Rica and Ohio. Ecol. Eng..

[36] Wang X.W., Song C.C., Sun X.X., Wang J.Y., Zhang X.H., Mao R. (2013). Soil carbon and nitrogen across wetland types in discontinuous permafrost zone of the Xiao Xing’an Mountains, northeastern China. Catena.

[37] Wang H., Wang R.Q., Yu Y., Mitchell M.J., Zhang L.J. (2011). Soil organic carbon of degraded wetlands treated with freshwater in the Yellow River Delta, China. J. Environ. Manage..

[38] Moore T.R., Dalva M. (1993). The influence of temperature and water-table position on carbon-dioxide and methane emissions from laboratory columns of peatland soils. Eur. J. Soil Sci..

[39] Keith H., Jacobsen K.L., Raison R.J. (1997). Effects of soil phosphorus availability, temperature and moisture on soil respiration in Eucalyptus pauciflora forest. Plant Soil.

[40] Reth S., Reichstein M., Falge E. (2005). The effect of soil water content, soil temperature, soil pH-value and the root mass on soil CO_2_ efflux-A modified model. Plant Soil.

[41] Sauze J., Ogee J., Maron P.A., Crouzet O., Nowak V., Wohl S., Kaisermann A., Jones S.P., Wingate L. (2017). The interaction of soil phototrophs and fungi with pH and their impact on soil CO_2_, (COO)-O-18 and OCS exchange. Soil Biol. Biochem..

[42] Søvik A.K., Augustin J., Heikkinen K., Huttunen J.T., Necki J.M., Karjalainen S.M., Klove B., Liikanen A., Mander U., Puustinen M. (2006). Emission of the greenhouse gases nitrous oxide and methane from constructed wetlands in Europe. J. Environ. Qual..

[43] Hernandez M.E., Mitsch W.J. (2006). Influence of hydrologic pulses, flooding frequency, and vegetation on nitrous oxide emissions from created riparian marshes. Wetlands.

[44] Vermue A., Philippot L., Munier-Jolain N., Henault C., Nicolardot B. (2013). Influence of integrated weed management system on N-cycling microbial communities and N_2_O emissions. Plant Soil.

[45] Teepe R., Brumme R., Beese F. (2000). Nitrous oxide emissions from frozen soils under agricultural, fallow and forest land. Soil Biol. Biochem..

[46] Song C.C., Zhang J.B., Wang Y.Y., Wang Y.S., Zhao Z.C. (2008). Emission of CO_2_, CH_4_ and N_2_O from freshwater marsh in northeast of China. J. Environ. Manage..

[47] Yu J.B., Liu J.S., Wang J.D., Sun W.D., Patrick W.H., Meixner F.X. (2007). Nitrous oxide emission from Deyeuxia angustifolia freshwater marsh in Northeast China. Environ. Manag..

[48] Dhondt K., Boeckx P., Hofman G., Van Cleemput O. (2004). Temporal and spatial patterns of denitrification enzyme activity and nitrous oxide fluxes in three adjacent vegetated riparian buffer zones. Biol. Fert. Soils.

[49] Li C.F., Cao C.G., Wang J.P., Zhan M., Yuan W.L. (2009). Ahmad, S. Nitrous oxide emissions from wetland rice–duck cultivation systems in southern China. Arch. Environ. Con. Toxicol..

[50] Chen H., Yuan X.Z., Gao Y.H., Wu N., Zhu D., Wang J.X. (2010). Nitrous Oxide Emissions from Newly Created Littoral Marshes in the Drawdown Area of the Three Gorges Reservoir, China. Water Air Soil Poll..

[51] He R.X., Jin H.J., Chang X.L., Wang Y.P., Wang L.Z. (2018). Freeze-thaw processes of active-layer soils in the Nanweng’he River National Natural Reserve in the Da Xing’anling Mountains, northern Northeast China. Sci. Cold Arid Reg..

[52] Liu P., Ren C.Y., Wang Z.M., Zhang B., Chen L. (2018). Assessment of the eco-environmental quality in the Nanweng River Nature Reserve, Northeast China by remote sensing. J. Appl. Ecol..

[53] Jiang H.Y., Zhao Y.S., Chen X.W., Li W.H., Zhu W.C., Lv W.B., Li X.P. (2007). Research on soil hydrology characteristics of some main forest type in south part of Daxing’anling. J. Soil Water Conserv..

[54] Lang H.Q. (1999). Chinese Wetlands Vegetations.

[55] Hedges J.I., Stern J.H. (1984). Carbon and nitrogen determinations of carbonate-containing solids. Limnol. Oceanogr..

[56] Gullón B., Yáñez R., Alonso J.L., Parajó J.C. (2008). L-Lactic acid production from apple pomace by sequential hydrolysis and fermentation. Bioresour. Technol..

[57] Song C.C., Wang Y.S., Wang Y.Y., Zhao Z.C. (2006). Emission of CO_2_, CH_4_ and N_2_O from freshwater marsh during freeze–thaw period in Northeast of China. Atmos. Environ..

